# Intrinsic subtypes and therapeutic decision-making in hormone receptor-positive/HER2-negative metastatic breast cancer with visceral crisis: A case report

**DOI:** 10.3389/fonc.2022.1009352

**Published:** 2022-11-08

**Authors:** Francesco Schettini, Elia Seguí, Benedetta Conte, Esther Sanfeliu, Blanca Gonzalez-Farre, Pedro Jares, Sergi Vidal-Sicart, Sergi Ganau, Isaac Cebrecos, Fara Brasó-Maristany, Montserrat Muñoz, Aleix Prat, Maria Vidal

**Affiliations:** ^1^ Medical Oncology Department, Hospital Clinic of Barcelona, Barcelona, Spain; ^2^ Translational Genomics and Targeted Therapies in Solid Tumors, August Pi I Sunyer Biomedical Research Institute (IDIBAPS), Barcelona, Spain; ^3^ Faculty of Medicine and Health Sciences, University of Barcelona, Barcelona, Spain; ^4^ Department of Pathology, Biomedical Diagnostic Center, Hospital Clinic of Barcelona, Barcelona, Spain; ^5^ Department of Nuclear Medicine, Diagnosis Imaging Center, Hospital Clinic of Barcelona, Barcelona, Spain; ^6^ Department of Radiology, Diagnosis Imaging Center, Hospital Clinic of Barcelona, Barcelona, Spain; ^7^ Clinic Institute of Gynecology, Obstetrics and Neonatology, Hospital Clinic of Barcelona, Barcelona, Spain; ^8^ Breast Cancer Unit, Institute of Oncology Barcelona (IOB) – Quirónsalud, Barcelona, Spain

**Keywords:** intrinsic subtype, HER2-enriched, ribociclib, metastatic breast cancer, hormone receptor (HR), HER2-negative, visceral crisis, CDK4/6-inhibitors

## Abstract

**Background:**

CDK4/6 inhibitors (CDKi), namely, palbociclib, ribociclib, and abemaciclib, combined with either an aromatase inhibitor (AI) or fulvestrant are the standard first/second line for hormone receptor-positive(HR+)/HER2-negative(neg) metastatic breast cancer (MBC). However, the choice of one specific CDKi is arbitrary and based on the physician’s experience with the drug, toxicity profile, and patient’s preferences, whereas biomarkers for optimal patient selection have not been established so far. Moreover, upfront chemotherapy is still recommended in case of clinical presentation with visceral crisis, despite no evidence of superior benefit for chemotherapy regimens against CDKi-based regimens. Recent correlative biomarker analyses from pivotal trials of palbociclib and ribociclib showed that HR+/HER2-neg MBC might respond differently according to the molecular intrinsic subtype, with Luminal A and B tumors being sensitive to both CDKi, Basal-like being insensitive to endocrine therapy, irrespective of CDKi, and HER2-enriched tumors showing a benefit only with ribociclib-based therapy.

**Clinical case:**

We hereby present a paradigmatic clinical case of a woman affected by a relapsed HR+/HER2-neg MBC with bone and nodal lesions, presenting with a visceral crisis in the form of lymphangitis carcinomatosis and diagnosed with a molecularly HER2-enriched tumor, successfully treated with upfront ribociclib + fulvestrant. The patient experienced a complete symptomatic and radiologic remission of the lymphangitis with a partial response as best response, according to RECIST 1.1 criteria. The progression-free survival (PFS) was of 20 months, in line with the median PFS observed in the ribociclib + fulvestrant pivotal trial, where, however, patients with visceral crisis had been excluded.

**Conclusions:**

This clinical case confirms in the real-world setting that non-luminal subtypes can be found in HR+/HER2-neg disease and may have potential therapeutic implications in the metastatic setting. It also questions the recommendation of upfront chemotherapy in the case of a visceral crisis in the era of CDKi-based regimens. These issues merit further evaluation in prospective and larger studies.

## Introduction

CDK4/6 inhibitors (CDKi), namely, palbociclib, ribociclib, and abemaciclib, combined with either an aromatase inhibitor (AI) or fulvestrant, are the standard first/second line for hormone receptor-positive(HR+)/HER2-negative(neg) metastatic breast cancer (MBC) (1–3). The recommendation is based on unprecedented progression-free survival (PFS) improvements, accompanied by a clinically relevant benefit in overall survival (OS), especially with ribociclib, never observed before with standard chemotherapy (CT) and ET regimens (4–10). However, the addition of a CDKi to ET is associated with more adverse events (AEs), compared with ET alone, as well as significantly higher costs (11–13). Unfortunately, no clear biomarker exists to help identify patients most benefiting from a CDKi and which one of the three should be the most proper for each single patient, although intrinsic subtypes (IS) might fill the gap (14, 15). Additionally, none of the CDKi trials included patients with visceral crisis (i.e., a short-term life-threatening clinical presentation of the metastatic disease); therefore, current guidelines still recommend the use of upfront CT in this clinical scenario (1–3). Here, we describe a paradigmatic case of a patient affected by a biologically aggressive HER2-enriched HR+/HER2-neg MBC with visceral crisis, treated successfully with first-line ribociclib+fulvestrant.

## Case report

**Figure 1:**
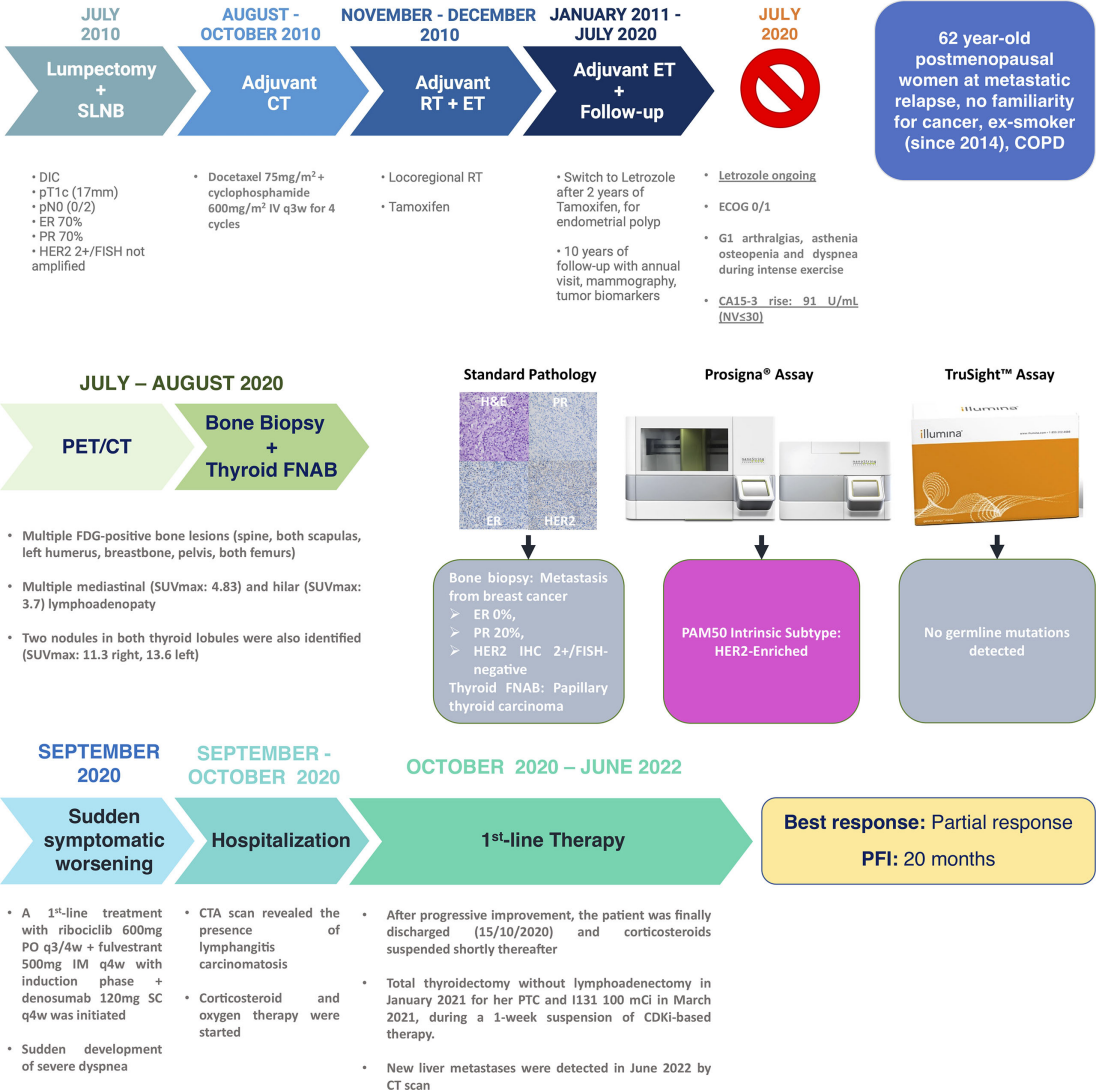
Case report timeline and clinicopathological details. Legend. Representative breast cancer pathology images from the patient’s bone biopsy are courtesy of Dr. Esther Sanfeliu (Pathology Department, Hospital Clinic of Barcelona). DIC, ductal invasive carcinoma; SLNB, sentinel lymph-node biopsy; CT, chemotherapy; IV, intravenous; IM, intramuscular; PO, *per os*; q3w, every 3 weeks; q3/4w, every 3 weeks out of 4; q4w, every 4 weeks; ET, endocrine therapy; RT, radiotherapy; NV, normal value; FISH, fluorescence *in situ* hybridization; COPD, chronic obstructive pulmonary disease; SUV, standard uptake volume; FNAB, fine needle aspiration biopsy; ER, estrogen receptor; PR, progesterone receptor; H&E, hematoxylin and eosin; CTA, computed tomography angiography; CDKi, CDK4/6 inhibitor; CT, computed tomography; PFI, progression-free interval; SC, subcutaneous and PTC, papillary thyroid carcinoma.

In July 2010, a 52-year-old premenopausal woman with chronic obstructive pulmonary disease due to smoking underwent lumpectomy+sentinel lymph-node biopsy at the Hospital Clinic of Barcelona (HCB), for an HR+/HER2-neg stage I BC of ductal histology. She subsequently received adjuvant CT, radiotherapy, and ET with tamoxifen, as of January 2011. After 2 years, the woman was switched to the AI letrozole for the development of endometrial polyps. By having transitioned to a postmenopausal status, a GnRH analogue was omitted. After the first 5 years of ET, it was decided to continue letrozole up to 10 years (**Figure 1**).

**Figure 2:**
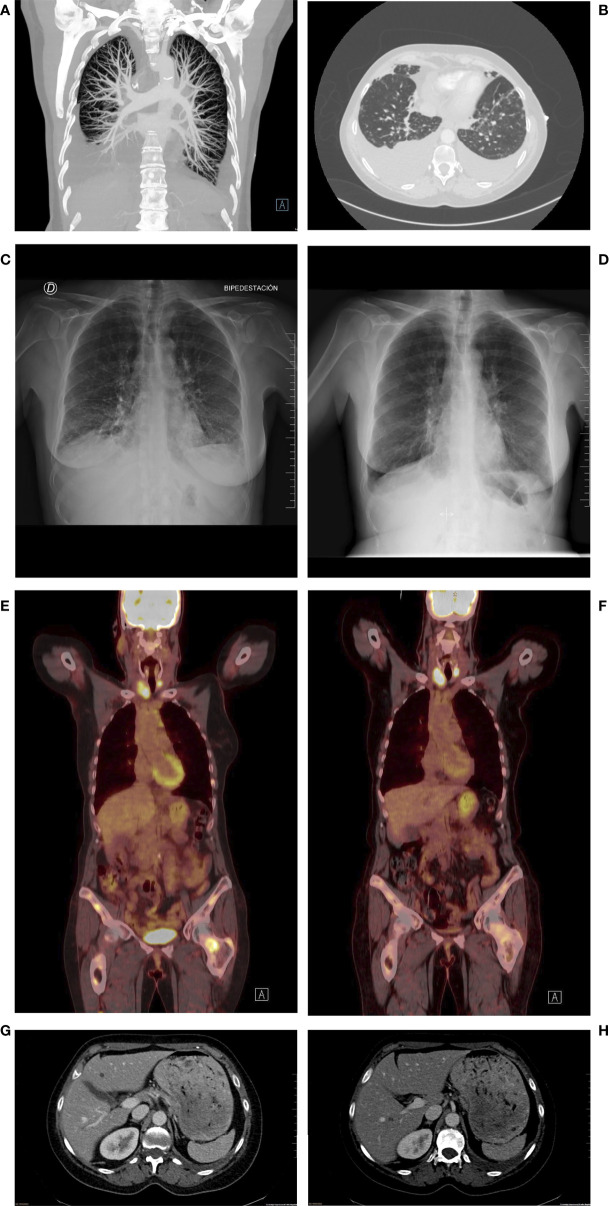
Representative images from the most relevant radiologic assessments. **(A, B)** CT Angiography at hospitalization showing signs of lymphangitis carcinomatosis. **(C, D)**Chest X-ray pre **(C)** and post **(D)** hospitalization showing the disappearance of lymphangitis signs and considerable reduction in pleural effusion. (**E, F)**: PET/CT imaging at metastatic relapse diagnosis **(E)** and at first reassessment **(F)** after first-line treatment start, showing SUV reduction in metastatic sites. (**G, H)**: CT scan at progression, showing two new hepatic lesions in the IV **(G)** and VI **(H)** segments.

In July 2020, a sudden rise in the CA15.3 tumor marker led to a radiologic tumor reassessment. A PET/CT detected multiple lesions with increased FDG uptake in bones and mediastinal and hilar lymph nodes (**Figure 2**). Since also two hypermetabolic nodules in both thyroid lobules were identified, a thyroid ultrasound-guided fine needle aspiration biopsy was undertaken, along with a bone biopsy. A tall-cell variant of a papillary thyroid carcinoma (PTC) was diagnosed in the thyroid, but the bone lesion was a metastasis from BC, with estrogen receptor (ER) and progesterone receptor (PR) expression levels of 0% and 20%, respectively, and no HER2 amplification/overexpression (**Figure 1**). Because of the double tumor diagnosis, the patient was then tested for the presence of genomic mutations in hereditary cancer predisposition genes with the assay Illumina TruSight™ Hereditary Cancer Panel (16). A Prosigna^®^ assay to identify the BC IS (17, 18) was carried out, as well. IS, namely, Luminal A, Luminal B, HER2-enriched, and Basal-like, are nosological entities characterized by peculiar gene expression patterns with a different natural history of the disease, prognosis, and response to treatments (17, 18). Prosigna^®^ is the standardized commercially available PAM50 assay, which is capable of detecting IS in patients’ fresh-frozen paraffin-embedded tumor samples, by assessing the expression of 50 genes (18–20). While TruSight^™^ could not identify any hereditary genomic alterations, Prosigna^®^ found the tumor to be molecularly HER2-enriched, a subtype which represents 10%–20% of all HR+/HER2-neg MBCs (21, 22).

After a multidisciplinary discussion, the treatment for the PTC was postponed and the combination of ribociclib+fulvestrant, with denosumab for bone lesions, was prescribed at the end of September 2020. Unfortunately, the patient suddenly developed dyspnea with minimal exertion during the first week of October 2020 and went to the emergency department of the HCB. At the physical examination, the patient presented with bilateral wheezing and right hypophonesis and a reduction in O_2_ saturation by pulse oximetry with limited physical exertion and high blood levels of NT-proBNP but negative ECG and cardiac ultrasonography. A chest X-ray identified signs of bilateral pleural effusion, and the patient was hospitalized. A subsequent CT angiography detected a subpleural nodule in the left lung compatible with a malignant pleural implant, along with conspicuous bilateral pleural effusions, peribronchovascular and interlobular septal thickening, centrilobular micronodules, and the already-detected mediastinal and hilar adenopathies, all signs suggestive for lymphangitis carcinomatosis (23) (**Figure 2**). The patient received a thoracocentesis, oxygen therapy, and intravenous corticosteroids. Despite being in a condition compatible with the definition of visceral crisis, namely, a severe potentially rapid life-threatening clinical condition (24–26), ribociclib and fulvestrant were not halted. Of note, we excluded ribociclib-induced interstitial lung disease (ILD) since this is a very rare side effect reported in 0.3% of cases (27). After a week, the patient could be discharged, continuing on-demand oxygen therapy at home along with oral corticosteroids, no longer required by the end of the same month. The patient experienced a partial response as best response, according to RECIST 1.1 criteria (28), with resolution of the lymphangitis and reduction in the number, dimension, and FDG uptake of nodal and bone lesions already at the December 2020 PET/CT (**Figures 1**, **2**). Ribociclib-based treatment was well tolerated, with only mild mucositis, skin dryness, and alopecia as AEs. The patient also underwent total thyroidectomy in January 2021 for her PTC and received I131 in March 2021, during a 1-week suspension of CDKi-based therapy. Liver disease progression was detected in June 2022, after a PFS of 20 months.

## Discussion

This clinical case is paradigmatic in several aspects. First, it highlights the potential relevance of BC IS for the therapeutic decision-making in HR+/HER2-neg MBC. At present, IS detection is not widespread in clinical practice for economic reasons, and surrogate subtype identification through IHC techniques assessing HR, HER2, and Ki67 levels guides therapeutic choices (29). In this case, the absence of ER and the low levels of PR, along with the relapse under an AI, pointed toward the presence of an endocrine-resistant tumor with a potential triple-negative breast cancer (TNBC)-like behavior, for which upfront CT might have been a viable therapeutic option (30–32). In this perspective, IS detection gave us the possibility to more properly assess the biologic nature of the disease. The identification of a HER2-enriched disease opened the opportunity to try an endocrine-based treatment approach. Notably, retrospective analyses in available patient samples from the PALOMA-2/-3 trials of palbociclib+letrozole and palbociclib+fulvestrant showed that non-Luminal subtypes (which were fewer than Luminals and dominated by the HER2-enriched) responded poorly to the palbociclib-based combinations, differently from Luminals A and B (33, 34). Conversely, a retrospective analysis on tumor samples from MONALEESA-2/-3/-7 trials showed that only patients with Basal-like disease (i.e., 3% of all cases) did not properly respond to ET, with/without ribociclib (14, 15). Moreover, HER2-enriched was the subtype that benefited most in terms of proportional improvements in PFS and OS (61% and 40% reduction in the instantaneous risk of progression or death, respectively) (14, 15).

Although this evidence is limited by its retrospective nature and methodologically heterogeneous IS detection (AIMS vs. research-based PAM50) (20), our real-world case seems to confirm ribociclib efficacy in the HER2-enriched disease. The explanation behind the differences in efficacy according to the CDK4/6 inhibitor is currently unknown; however, the differences in efficacy and toxicity seen in patients might be explained by the different potency of these drugs in inhibiting CDK4 versus CDK6 (35) and/or the different dosage used (higher for ribociclib). In any case, the currently ongoing phase III trial HARMONIA (NCT05207709) is randomizing patients with molecularly HER2-enriched HR+/HER2-neg MBC to receive ET with palbociclib or ribociclib. An exploratory cohort of 60 patients with Basal-like tumors will also be recruited to preliminarily assess upfront CT benefit in this context (36). Worthy of note is that no evidence on abemaciclib efficacy according to tumor IS has been produced so far, making its role unclear in this perspective.

A second aspect to highlight is that this report further confirms that BC IHC surrogate subtypes do not completely overlap with IS, as also previously shown (21, 22, 37). Within HR+/HER2-neg BC, the HER2-enriched subtype is found in 6%–20% of cases (21, 22) and does not differ much from that within clinical HER2-positive BC from a gene expression perspective, except for a lack of *ERBB2* overexpression and amplification (38). HER2-enriched diseases seem to be characterized by higher levels of *APOBEC3B*-mediated mutagenesis and immune infiltration compared with other IS and have proven to be more chemo-sensitive than HR+/Luminal A or B tumors (22, 38–40) but with worse prognosis (41).

Interestingly in our case, the 20-month PFS experienced with the combination of first-line ribociclib+fulvestrant confirmed the HER2-enriched sensitiveness to ribociclib and, notably, was in line with the median PFS observed in the pivotal MONALEESA-3 trial of ribociclib+fulvestrant, where patients with a visceral crisis were not included (42). In fact, this report also questions the recommendation to treat with CT all patients with visceral crisis. This recommendation, based on the idea that CT could provide more rapid and higher response rates (RR) than ET, was adopted in the pre-CDKi era. However, a recent network meta-analysis comparing all ET and CT with/without targeted agents for the first/second line of HR+/HER2-neg MBC confirmed the significant superiority of CDKi-based regimens in terms of PFS and RR over standard ET and reported comparable efficacy and activity with the main currently available CT options (11). A similar result was subsequently observed in the PEARL phase III RCT of palbociclib+exemestane/fulvestrant vs. capecitabine (43). Moreover, an objective response with CDKi-based combinations is usually achieved quickly and within the first 3 months for the majority of responders (44, 45). Finally, a recent report on a retrospective dataset of patients with HR+/HER2-neg MBC showed that the use of upfront CDKi in the presence of visceral crisis was associated with a 5-month improvement in OS and a 2-year OS of 26.1% vs. 8.1%, compared with CT (46). Unfortunately, an RCT in the setting of visceral crisis comparing CDKi to CT will likely never be conducted. Nevertheless, all these results, including our experience from this case report, support the possibility to use CDKi also in this poor prognostic context, at least if patients present with a severe clinical condition that does not represent *per se* an obstacle to CDKi prescription. In fact, the pattern of visceral crisis rather than visceral crisis itself might guide the selection of the most proper treatment, according to systemic agents’ side effects and pharmacokinetic profile. For example, apart from the case hereby reported, patients with medullary infiltration and neutropenia/pancytopenia might be considered candidates to receive abemaciclib instead of other CDKi, since the former is characterized by lower rates of hematotoxicity than palbociclib/ribociclib and is potentially less myelodisruptive than standard CT, or, in moderate–severe liver dysfunction cases, considering the preferential liver metabolism of all the three approved CDKi, other agents which have been proven to be safely administrable in patients with impaired hepatic function, such as capecitabine or platinum agents (47), should be preferred.

To conclude, this case report confirms in the real-world setting that all the IS can be found in HR+/HER2-neg MBC, having potential therapeutic implications, with the HER2-enriched subtype being especially sensitive to ribociclib. It also questions the recommendation of upfront CT in the case of a visceral crisis in the era of CDKi-based regimens. These issues merit further evaluation.

## Data availability statement

The original contributions presented in the study are included in the article. Further inquiries can be directed to the corresponding authors.

## Ethics statement

Written informed consent was obtained from the individual(s) for the publication of any potentially identifiable images or data included in this article.

## Author contributions

Conception and design: FS and MV. Financial support: FS and AP. Administrative support: none. Provision of study materials or patients: all authors. Collection and assembly of data: all authors. Data analysis and interpretation: all authors. Manuscript writing: FS, MV, AP. Final approval of manuscript: all authors. Accountable for all aspects of the work: all authors. All authors contributed to the article and approved the submitted version.

## Acknowledgments

F.S. is the recipient of a European Society for Medical Oncology (ESMO) Fellowship – Translational and BBVA Foundation/Hospital Clinic of Barcelona Joan Rodés - Jose Baselga Advanced Research Contract in Oncology. A.P. is supported by a Breast Cancer Now—2018NOVPCC1294 grant, Breast Cancer Research Foundation-AACR Career Development Awards for Translational Breast Cancer Research 19-20-26-PRAT, Fundació La Marató TV3 201935-30 grant and RESCUER, funded by European Union's Horizon 2020 Research and Innovation Programme under Grant Agreement No. 847912. F.B-M. is supported by a Fundación Científica Asociación Española Contra el Cáncer - INVES21943BRAS grant. Any views, opinions, findings, conclusions, or recommendations expressed in this material are those solely of the authors and do not necessarily reflect those of funders/sponsors.

## Conflict of interest

AP declares no competing non-financial interests but reports advisory and consulting fees from Roche, Pfizer, Novartis, Amgen, BMS, Puma, Oncolytics Biotech, MSD, Guardant Health, Peptomyc, and Lilly; lecture fees from Roche, Pfizer, Novartis, Amgen, BMS, NanoString Technologies, and Daiichi Sankyo; and institutional financial interests from Boehringer, Novartis, Roche, NanoString, Sysmex Europa GmbH, Medica Scientia Innovation Research, SL, Celgene, Astellas, and Pfizer and shares ownership and a leadership role in Reveal Genomics, SL. MV declares consulting fees e.g., advisory boards from Roche, Novartis, and Daiichi Sankyo/AstraZeneca; support for attending meetings and/or travel from Roche, Novartis, and Daiichi Sankyo/AstraZeneca; and honoraria for presentations by Roche, Novartis, and Daiichi Sankyo/AstraZeneca FS declares honoraria for presentations/educational materials paid by Novartis..

The remaining authors declare that the research was conducted in the absence of any commercial or financial relationships that could be construed as a potential conflict of interest.

## Publisher’s note

All claims expressed in this article are solely those of the authors and do not necessarily represent those of their affiliated organizations, or those of the publisher, the editors and the reviewers. Any product that may be evaluated in this article, or claim that may be made by its manufacturer, is not guaranteed or endorsed by the publisher.
